# Under Pressure: Mechanical Stress Management in the Nucleus

**DOI:** 10.3390/cells5020027

**Published:** 2016-06-14

**Authors:** Néjma Belaadi, Julien Aureille, Christophe Guilluy

**Affiliations:** 1 Institut National de la Santé et de la Recherche Médicale, UMR 1087, l’institut du thorax, CNRS, UMR 6291, Université de Nantes, 44000 Nantes, France; nejma.belaadi@univ-nantes.fr; 2 Institut National de la Santé et de la Recherche Médicale, UMR 1209, Institute for Advanced Biosciences, CNRS UMR 5309, Université Grenoble Alpes, 38041 Grenoble, France; julien.aureille@inserm.fr

**Keywords:** mechanotransduction, nucleoskeleton, lamina, lamin, LINC, nucleus, mechanical stress

## Abstract

Cells are constantly adjusting to the mechanical properties of their surroundings, operating a complex mechanochemical feedback, which hinges on mechanotransduction mechanisms. Whereas adhesion structures have been shown to play a central role in mechanotransduction, it now emerges that the nucleus may act as a mechanosensitive structure. Here, we review recent advances demonstrating that mechanical stress emanating from the cytoskeleton can activate pathways in the nucleus which eventually impact both its structure and the transcriptional machinery.

## 1. Introduction

It has long been observed that mechanical forces play an important role in biology, perhaps most remarkably throughout the process of morphogenesis when tissue architecture appears to be shaped by mechanical loading [1,2]. However, it is only recently that the molecular mechanisms activated by mechanical stress are beginning to be elucidated. These recent advances were allowed by the emergence of new technologies to manipulate and measure mechanical tension at the molecular level, such as optical tweezers or atomic force microscopy [3,4] and more recently, the design of molecular “tension sensors” [5,6]. Additionally, the development of new surfaces with tunable mechanical or geometrical properties [7,8,9,10,11,12] enabled us to culture cells in a controlled microenvironment, thus extending the possibility to explore their mechanics. Within the last 20 years, these tools have yielded numerous findings, demonstrating that changes in the mechanical properties of the cellular environment can deeply affect cell behavior and fate[10,13,14,15]. Mechanical tension, whether it is generated by the cell’s cytoskeleton or externally applied to the cell surface, has been shown to regulate cell division and growth [8,16] or orient the transcriptional machinery and drive cell differentiation [10,14]. Interestingly, these mechanical stimuli can have an antagonistic effect on the cellular behavior. The quintessential example of this dual effect is certainly the impact of shear stress on endothelial cells, which depends on blood flow magnitude, frequency and direction [17,18,19]. Whereas low and oscillatory shear stress will promote inflammation and atherosclerosis development, high and unidirectional shear stress prevents inflammation and plaque formation [18]. 

## 2. Sensing and Responding to Mechanical Force

In parallel to the emerging importance of mechanical signals in biology, scientists have questioned the molecular mechanisms which allow cells to “sense” and transduce mechanical force into biochemical pathways. This has led to the identification of molecular mechanisms that coordinately transmit and transduce mechanical stress in order to trigger an appropriate cellular response [19]. Typical mechanotransduction mechanisms involve proteins whose conformation can be regulated by mechanical tension, ultimately affecting its post-translational modification (PTM), interaction or subcellular localization. Among the first candidates identified as mechanosensors were cell membrane proteins which directly experience stress applied to the cell surface, such as mechanosensitive ion channels whose activity are regulated by membrane tension [20]. In this context, the protein complexes that constitute adhesion with the extracellular matrix (ECM) or with neighboring cells have been shown to play an important role in mechanotransduction [21,22,23,24,25]. 

Mechanical tension can affect not only components of the cell surface, but it can also regulate molecular processes within the nucleus, such as gene expression or DNA damage [14,26,27,28,29]. Both externally applied and internally generated mechanical forces have been shown to regulate the gene expression patterns [27]. Two main possibilities of mechanical stress transduction to the nucleus have been proposed: (1) Tension may signal to the nucleus via a cascade of biochemical pathways that involve translocation of cytoplasmic components into the nucleus; (2) another possibility is that mechanical stress may be transmitted to the nucleus where it activates signaling pathways to regulate gene expression [30]. Multiple molecular relays have been found to shuttle from the cytoplasm to the nucleus in response to mechanical stress [31,32,33], such as proteins containing the Lin11-Is1-Mec3 (LIM) domain [34] or Yes-associated protein (YAP) and transcriptional coactivator with PDZ-binding motif (TAZ) [31], indicating that the first transduction modality plays a major role. However, recent evidences indicate that a mechanotransduction mechanism exists within the nucleus and may contribute to control cell fate and phenotype [35,36,37]. 

## 3. Mechanical Stress Transmission to the Nucleus

The possibility that mechanical stress can be transmitted from the cell surface to the nucleus was first demonstrated in a seminal work from Donald Ingber’s group [38]. Using beads coated with integrin ligand and a glass micropipette, they applied tensional forces to the cell surface adhesion of endothelial cells and observed nuclear envelope (NE) distortion. Interestingly, Maniotis and colleagues observed that both actin and intermediate filaments participate to stress transmission. These results indicated for the first time that cell surface integrins were connected with the nucleus [38]. 

**Figure 1:**
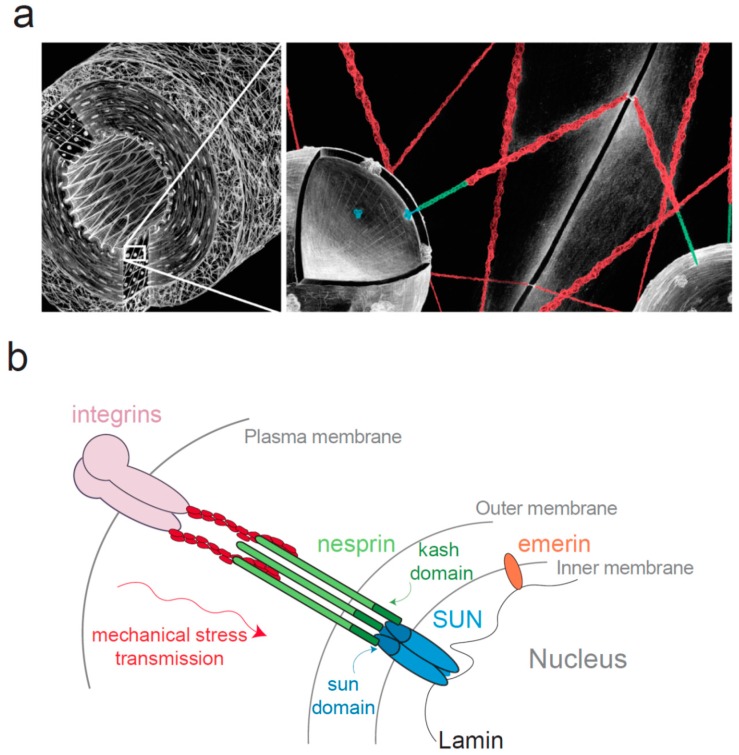
The Linker of Nucleoskeleton and Cytoskeleton (LINC) complex connects the nucleus to the cytoskeleton. (**a**) Diagram of a blood vessel (left panel) at two different scales, demonstrating the mechanical continuum that exists between the cell surface adhesion and the nucleus; (**b**) Schematic representation of the LINC complex. This complex consists in SUN proteins anchored in the inner nuclear membrane (INM) and nesprins anchored in the outer nuclear membrane (ONM). SUN domain was shown to organize in a trimeric fashion to bind three KASH peptides [50,51].

Whereas the adhesion proteins that connect the cytoskeleton with integrins have been extensively studied [39,40,41,42], the protein responsible for the attachment of the cytoskeleton to the nucleus were identified only recently [43,44,45] and belong to the Linker of Nucleoskeleton and Cytoskeleton (LINC) complex [43,44] (Figure 1). This complex consists in SUN (Sad1 and UNC-84) proteins anchored in the inner nuclear membrane (INM) and nesprin (nuclear envelope spectrin-repeat-containing proteins also called Syne, Myne or NUANCE) anchored in the outer nuclear membrane (ONM) [43,46,47,48]. As first observed with SUN1 [49], SUN proteins and nesprins interact together within the perinuclear space respectively through their SUN (Sad1 Unc-84 homology) domain highly conserved from fission yeast to mammals and KASH (Klarsicht, ANC-1, and Syne homology) domain, a short peptide of ~30 residues associated with a transmembrane domain (Figure 1). The crystal structure of SUN/KASH interaction has been recently determined and SUN domain was shown to organize in a trimeric fashion to bind three KASH peptides [50,51]. The stability of this interaction is essential for force transmission, and depends partly on a covalent disulfide bond of conserved cysteines between SUN2 and KASH2 [52]. SUN proteins exist in five isoforms in humans (SUN1 to SUN5). SUN1 and SUN2 are widely expressed [49] while SUN3, SUN4 also known as SPAG4 (Sperm Associated Antigen 4) and SUN5 also known as SPAG4L (SPAG4-Like) have a more restricted expression, they are almost exclusively found in testes [53,54,55,56]. SUN proteins are linked to lamins [46] and chromatin in the nucleoplasm [57,58]. Recent work showed that SUN1 and SUN2 co-depletion slows down the removal of chromatin during nuclear envelope breakdown, SUN proteins facilitate then mitotic progression [58]. This interaction with DNA might suggest that SUN proteins may participate in gene transcription regulation. Four nesprin isoforms have been identified in humans (nesprin-1 to nesprin-4), themselves with multiple splice isoforms able to link cytoskeletal components [59]. Nesprin-1 and -2 bind to F-actin [48,60] and to microtubules motors such as kinesin, dynein and meckelin in the cytoplasm [61,62]. Nesprin-3 interacts with plectin, which binds intermediate filaments and integrin α6β4 in keratinocytes [63,64,65,66]. Nesprin 4 can connect with microtubules via kinesin [67]. Interestingly, not all nesprins are found anchored in the ONM and some isoforms such as nesprin-1α or nesprin-2 are located in the INM where they have been shown to interact directly with lamins and emerin [68,69]. 

Because the LINC complex proteins are anchored in the NE, they were widely described as contributing to nuclear organization. Their silencing causes nuclei distortion, NE deformations and blebbing in different cell types [70,71,72]. Depleting SUN in *Caenorhabditis elegans*, Starr and colleagues observed that most of the cells had normal NE architecture and only muscle cells displayed NE spacing abnormalities, suggesting that functional LINC may only be necessary for maintaining NE width in cells subjected to high mechanical forces [73]. 

## 4. Mechanical Stress Experienced by the Nucleus

As a consequence of its LINC-mediated anchorage to the NE, the cytoskeleton transmits mechanical stress emanating from the ECM or generated by molecular motors to the nucleus. For example, tensional forces are applied to nesprin-2G by the large array of actomyosin-based TAN (transmembrane actin-associated nuclear) lines to allow nuclear movements. Gundersen and colleagues demonstrate that TAN lines promote centrosome reorientation in migrating cells [74], by driving rearward nuclear movement and positioning the centrosome between the nucleus and the leading edge of the cell [74,75,76,77]. It was recently confirmed that myosin-generated tension is applied to nesprin2-G by using a fluorescence resonance energy transfer (FRET)-based tension biosensor developed with a shortened version of nesprin-2G, although the exact amount of force experiences by the LINC complex remains to be determined [78]. 

**Figure 2:**
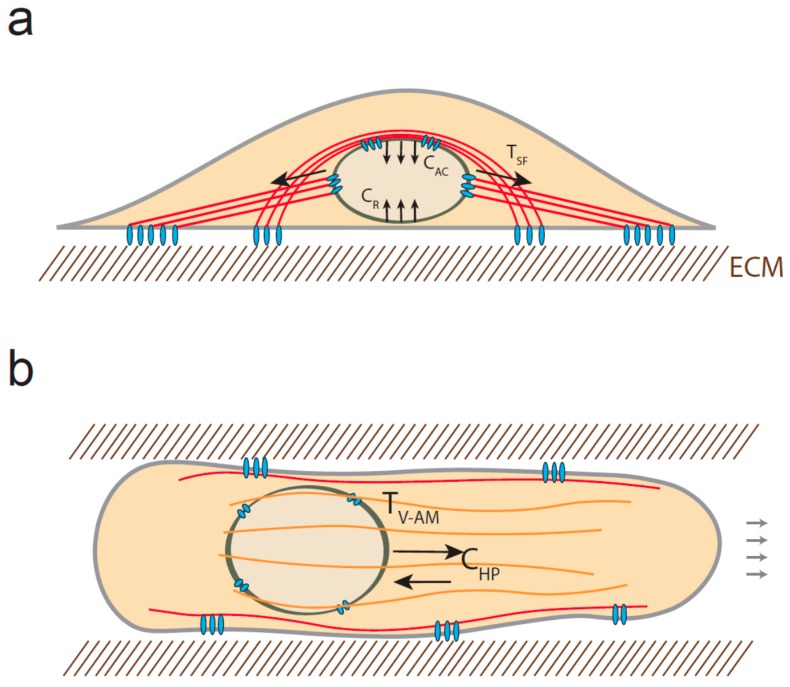
Mechanical stress experienced by the nucleus. (**a**) Diagram of a stationary cell. When cells are cultured on 2D surfaces, the nucleus can be subjected to tensional forces emanating from stress fibers (T_SF_) and compressive forces due to the actin cap (C_AC_) structures and the resistance of the surface (C_R_). The red solid lines represent the actin filaments; (**b**) In 3D, cells may also experience both tension, generated by vimentin-associated actomyosin filaments (orange structures) (T_V-AM_) and compression resulting from the high pressure of the anterior compartment (C_HP_).

Actin filaments can also apply compressive force to the nucleus. When cells are cultured on a flat surface, a subset of contractile actin filament bundles cover the nucleus and form the so-called actin cap [79]. These filaments control nucleus rotation and translocation via their attachment to the nuclear envelope through KASH-SUN interaction, which thus promotes cell repolarization and migration [80]. As a consequence, the nucleus is compressed between the actin cap structures and the substrate, resulting in a compressive force whose amount depends on the rigidity of the substrate [36] (Figure 2a). 

Interestingly, the nucleus may also be subjected to both compressive and tensile forces when cells migrate in 3D. Petrie and colleagues observed that the nucleus separates an anterior high-pressure compartment from a low pressure compartment behind the nucleus [81]. They showed that both myosin and vimentin interact with nesprin-3, indicating that myosin-generated tension may be transmitted to intermediate filaments and LINC complex in order to pull the nucleus forward and increase compartmentalized pressure. As a result, one could anticipate that in addition to the tension applied to nesprin 3, the anterior side of the nucleus may be subjected to compressive force resulting from the pressure gradient (Figure 2b). Additionally, it has recently been shown that the nucleus can be substantially compressed when cells migrate in confining environment [27,82]. Two independent studies indicate that the highly strained NE can rupture and get repaired at the leading tip of the nucleus as it progresses into small constriction [27,82]. Interestingly, lamin A/C depletion increases NE ruptures occurrence, whereas increased-lamin A/C expression limits nuclear deformation and prevents migration through small pores. This indicates that lamin expression and organization may determine the capacity of cells to migrate in 3D [83,84]; however, it is not known if lamin A/C can dynamically remodel in response to compression. 

## 5. Nuclear Response to Mechanical Force

Various methods have been developed to apply mechanical stress to nuclei and measure their response in order to decipher their mechanical properties [35,37,85,86,87,88,89], using either nuclei in intact cells or isolated nuclei. These experimental approaches showed that both lamina-based nucleoskeleton and chromatin contribute to the mechanical properties of nuclei [88,90]. While the nucleoskeleton displays viscoelastic mechanical properties, the chromatin has been shown to be viscous and has liquid-like behavior [88,90]. The nuclear lamina represents a protein meshwork composed of type-A and type-B lamins that form apparently distinct networks [91,92,93]. Whereas at least one type-B lamin is expressed in every mammalian cells, lamin A/C levels can vary depending on the cell type [91]. Lamin A/C is a major contributor to NE mechanical properties [35,88,90] and depletion of lamin A/C increase nuclear deformability in response to mechanical stress [35,88,94]. Depletion of lamin-partners which connect the lamina to the LINC complex or to the envelope, such as titin, αII-spectrin [95] or emerin [35] has also been shown to affect nuclear strain. This indicates that local nuclear strain may depend not only on lamin A/C level, but also on its connection to other nucleoskeletal elements which experience mechanical stress, whether it is the NE or the LINC complex.

Investigating the nuclear response to force, some recent studies observed that the nucleoskeleton can dynamically remodel in response to mechanical stress, indicating that the nucleus behaves like a material that can adjust its own mechanical properties depending on the mechanical load [35,36,37,96]. The Disher lab showed that lamin A/C level matches with tissue elasticity in mice and human cells, as a result of lamin dephosphorylation, subsequent stabilization, and vitamin A/retinoic acid-dependent effect on lamin transcription [37]. They showed that this mechanism controls gene expression and participates in matrix-directed stem cell lineage determination. Additionally, they observed that application of shear stress to isolated nuclei induces a decrease in lamin phosphorylation at Ser390 [37], indicating that lamin A/C conformation may be sensitive to mechanical stress. Consistent with this, Vogel and colleagues showed that lamin A/C undergoes a conformational change when subjected to compressive force by using an antibody that recognizes a conformational epitope [36]. It will be interesting to test if this conformational change can affect lamin phosphorylation or stability. Applying tension to nesprin 1 in isolated nuclei, we recently found that emerin mediates a nuclear stiffening in response to pulses of force [35]. We observed that emerin phosphorylation triggers a rearrangement of the LINC complex that strengthens the connection between the LINC and lamin A/C, thus limiting nuclear deformation. Remarkably, the response was very similar to what has been described when tension was applied to cell surface adhesion [97,98]. Philip and Dahl showed that lamins are reorganized in response to shear stress and nuclear periphery, which may also be due to reinforcement of the connection between LINC and lamin A/C [99]. Whether emerin is necessary for this response remains to be determined. Interestingly, the cytoplasmic protein FHOD1 has been shown to strengthen the interaction between actin and nesprin-2G [77,100] in response to tension, suggesting that a coordinated strengthening of the cytoskeletal elements may occur from the adhesion to the nucleus in response to tension. However, one could anticipate that this stiffening response is more likely limited, especially in tissues subjected to frequent stretch, suggesting that counteracting mechanisms may exist to limit nuclear stiffness or decrease its connection to the LINC complex depending on the mechanical constraint of the environment. 

## 6. Conclusions

Recent work indicates that the nucleus may act as a mechanosensitive structure, whose nucleoskeleton can dynamically remodel in response to changes in the mechanical properties of the cellular microenvironment. This dynamic reorganization hinges on nuclear mechanotansduction mechanisms and the initial mechanosensor(s) that triggers this response remains to be identified [93,96,101]. Lamin A/C conformation is sensitive to mechanical stress, but we can speculate that different mechanosensors may exist and be independently activated, whether mechanical stress is applied to the LINC complex or to the overall NE. It will be crucial to measure the amount of stress experienced by the nucleus *in vivo*, in order to test the relevance of such mechanisms. Whether these nuclear mechanotransduction mechanisms can control gene expression while the nucleus experiences force, remains also to be clarified. Certainly, depletion or mutation of nucleoskeletal components can impact transcription such as SRF or YAP/TAZ [35,37,102,103], but further investigations are required to understand how dynamic nucleoskeletal remodeling can affect transcription factor activity or chromatin organization.
